# Comment on "Molecular monitoring by CDKN2A/p16INK4A and RB1 gene methylation in breast cancer"

**DOI:** 10.1590/1806-9282.20241031

**Published:** 2025-03-17

**Authors:** Huaxiao Tang, Lianlian Song

**Affiliations:** 1 Qingdao University, Weihai Second Affiliated Hospital (Weihai Maternal and Child Health Hospital), Department of Pathology – Weihai, China.

Dear Editor,

We were very pleased to read the study by Queiroz^1^ and his colleagues, in which they revealed that comprehensive analysis of the methylation status of two pivotal genes—CDKN2A/p16INK4A (cyclin-dependent kinase inhibitor 2A) and RB1 (retinoblastoma transcriptional corepressor 1)—can detect changes in methylation patterns before any visible sign of cancer appears in breast tissues and could help predict the recurrence of malignant breast tumors. This study provides in-depth insight for preventing chronic kidney disease and presents a novel approach for monitoring breast cancer patients through the assessment of methylation in circulating free deoxyribonucleic acid (DNA). However, some views should be raised in my views.

From the results in Tables 1 and 2, we found that the circulating free DNA methylation rates of these two genes—2/15 and 1/15, respectively—were very low. Therefore, monitoring breast cancer by assessing circulating cell-free DNA methylation is not possible.

Factors, educational level, and lifestyle factors were not available in this study.

Additionally, the detailed exclusion criteria for the healthy volunteers were not provided. What is more, the participants assessed in this study were mostly subjects across a large age span.

The design of the study was flawed, as all patients had confirmed breast cancer. The level of cell-free DNA methylation in all patients was significantly low both times after surgery. If cell-free DNA methylation is to be used for early cancer screening, it should first be dynamically monitored before the occurrence of breast cancer. This study would be more reasonable if conducted as a cohort study.
